# Peste des Petits Ruminants Virus Infection at the Wildlife–Livestock Interface in the Greater Serengeti Ecosystem, 2015–2019

**DOI:** 10.3390/v13050838

**Published:** 2021-05-06

**Authors:** Bryony A. Jones, Mana Mahapatra, Daniel Mdetele, Julius Keyyu, Francis Gakuya, Ernest Eblate, Isaac Lekolool, Campaign Limo, Josephine N. Ndiwa, Peter Hongo, Justin S. Wanda, Ligge Shilinde, Maulid Mdaki, Camilla Benfield, Krupali Parekh, Martin Mayora Neto, David Ndeereh, Gerald Misinzo, Mariam R. Makange, Alexandre Caron, Arnaud Bataille, Geneviève Libeau, Samia Guendouz, Emanuel S. Swai, Obed Nyasebwa, Stephen L. Koyie, Harry Oyas, Satya Parida, Richard Kock

**Affiliations:** 1 Royal Veterinary College, University of London, Hawkshead Lane, North Mymms, Hatfield AL9 7TA, UK; cbenfield@rvc.ac.uk (C.B.); rkock@rvc.ac.uk (R.K.); 2 The Pirbright Institute, Ash Road, Pirbright, Surrey GU24 0NF, UK; mana.mahapatra@pirbright.ac.uk (M.M.); krupali.parekh@pirbright.ac.uk (K.P.); martinneto@hotmail.com (M.M.N.); satya.parida@pirbright.ac.uk (S.P.); 3 SACIDS Foundation for One Health, Sokoine University of Agriculture, P.O. Box 3297 Morogoro, Tanzania; mdeteled@gmail.com (D.M.); gerald.misinzo@sacids.org (G.M.); mirrichy@gmail.com (M.R.M.); 4 Tanzania Wildlife Research Institute, P.O. Box 661 Arusha, Tanzania; julius.keyyu@gmail.com (J.K.); eblate.ernest@gmail.com (E.E.); shamanchejustin@gmail.com (J.S.W.); ligge.shillinde@tawiri.or.tz (L.S.); mluziga@yahoo.co.uk (M.M.); 5 Kenya Wildlife Service, P.O. Box 40241-00100 Nairobi, Kenya; gakuya@kws.go.ke (F.G.); lekolool@kws.go.ke (I.L.); limocampaign@gmail.com (C.L.); peterh@kws.go.ke (P.H.); dndeereh@kws.go.ke (D.N.); 6 Private Consultant, P.O. Box 42385-00100 Nairobi, Kenya; josephinendiwa@gmail.com; 7 Food and Agriculture Organization of the United Nations (FAO), Viale delle Terme di Caracalla, 00153 Rome, Italy; 8 Animals, Health, Territories, Risks and Ecosystem (ASTRE), University of Montpellier (UMR), Centre de Coopération Internationale en Recherche Agronomique pour le Développement (CIRAD), National Research Institute for Agriculture, Food and the Environment (INRAE), F-34398 Montpellier, France; alexandre.caron@cirad.fr (A.C.); arnaud.bataille@cirad.fr (A.B.); genevieve.libeau@cirad.fr (G.L.); samia.sahnoune_guendouz@cirad.fr (S.G.); 9 CIRAD, UMR ASTRE, RP-PCP, Maputo 01009, Mozambique; 10 Faculdade de Veterinária, Universidade Eduardo Mondlane, P.O. Box 257, Maputo 01009, Mozambique; 11 CIRAD, UMR ASTRE, F-34398 Montpellier, France; 12 Directorate of Veterinary Services, Ministry of Livestock and Fisheries, Mji wa Serikali Mtumba, Mtaa wa Ulinzi, P.O. Box 2870 Dodoma, Tanzania; esswai@gmail.com (E.S.S.); obedmalangu@gmail.com (O.N.); 13 Department of Agriculture, Livestock and Fisheries, Narok County Government, P.O. Box 898-20500 Narok, Kenya; steve.leshan@gmail.com; 14 Directorate of Veterinary Services, Ministry of Agriculture, Livestock and Fisheries, P.O. Box 30028-00100 Nairobi, Kenya; harryoyas@gmail.com

**Keywords:** PPR, epidemiology, transboundary animal disease, surveillance, eradication, wild animals, sheep, goats, Kenya, Tanzania

## Abstract

Peste des petits ruminants (PPR) is a viral disease of goats and sheep that occurs in Africa, the Middle East and Asia with a severe impact on livelihoods and livestock trade. Many wild artiodactyls are susceptible to PPR virus (PPRV) infection, and some outbreaks have threatened endangered wild populations. The role of wild species in PPRV epidemiology is unclear, which is a knowledge gap for the Global Strategy for the Control and Eradication of PPR. These studies aimed to investigate PPRV infection in wild artiodactyls in the Greater Serengeti and Amboseli ecosystems of Kenya and Tanzania. Out of 132 animals purposively sampled in 2015–2016, 19.7% were PPRV seropositive by ID Screen PPR competition enzyme-linked immunosorbent assay (cELISA; IDvet, France) from the following species: African buffalo, wildebeest, topi, kongoni, Grant’s gazelle, impala, Thomson’s gazelle, warthog and gerenuk, while waterbuck and lesser kudu were seronegative. In 2018–2019, a cross-sectional survey of randomly selected African buffalo and Grant’s gazelle herds was conducted. The weighted estimate of PPRV seroprevalence was 12.0% out of 191 African buffalo and 1.1% out of 139 Grant’s gazelles. All ocular and nasal swabs and faeces were negative by PPRV real-time reverse transcription-polymerase chain reaction (RT-qPCR). Investigations of a PPR-like disease in sheep and goats confirmed PPRV circulation in the area by rapid detection test and/or RT-qPCR. These results demonstrated serological evidence of PPRV infection in wild artiodactyl species at the wildlife–livestock interface in this ecosystem where PPRV is endemic in domestic small ruminants. Exposure to PPRV could be via spillover from infected small ruminants or from transmission between wild animals, while the relatively low seroprevalence suggests that sustained transmission is unlikely. Further studies of other major wild artiodactyls in this ecosystem are required, such as impala, Thomson’s gazelle and wildebeest.

## 1. Introduction

Peste des petits ruminants (PPR) is a highly infectious disease of goats and sheep that is widespread across Africa, the Middle East and Asia. The disease has also occurred in some captive and free-ranging wild artiodactyls. It is caused by PPR virus (PPRV, *Small ruminant morbillivirus*), which is most closely related to rinderpest and measles viruses [1,2]. PPR disease has a severe impact on food security, livelihoods and small ruminant trade for livestock-keeping communities. It is an important transboundary animal disease that has been targeted for global eradication by the World Organisation for Animal Health (OIE) and the Food and Agriculture Organization of the United Nations (FAO) [3,4,5]. PPR disease has severely impacted critically endangered wild species such as the saiga antelope *(Saiga tatarica mongolica),* probably caused by virus of livestock origin, making it one of the important diseases of concern to wildlife conservation [6] with a potential impact on wildlife tourism revenue, adding further incentive for the elimination of this disease from livestock.

The main route of PPRV transmission is by direct contact between infectious and susceptible animals [1,2]. Therefore, important mechanisms for animal-to-animal virus transmission are the sharing of water and grazing resources between domestic flocks and with wild animals, through transhumant and nomadic flock movements, as well as small ruminant trade networks [7].

### 1.1. Wildlife and Peste des Petits Ruminants

A wide range of wild artiodactyl species are susceptible to PPRV infection, based on the serological detection of PPRV antibodies, and outbreaks of clinical disease in Asia have been diagnosed as PPR on the basis of the molecular detection of PPRV. In most of these cases, it is likely that there was spillover of the virus from nearby populations of infected domestic small ruminants leading to sporadic wildlife epidemics and possible cross-species transmission between wild species, while spillback from wildlife to livestock has not been reported [6,8,9,10,11,12,13,14]. 

In Africa, the only reports of antigenic or molecular confirmation of PPR disease in wildlife species to date were in Sudan [12,15]. This is surprising, considering the large populations of PPRV susceptible wild species in Africa, and needs further investigation. It is possible that PPR disease has occurred in other free-ranging wild species in other regions of Africa, but has not been detected or has not been reported because of the remoteness of wild populations, limited capacity for wild animal disease surveillance and the fact that sick or dead wild animals could be removed by predators and scavengers before they are identified. The reports of PPR from Sudan were in Nile lechwe (*Kobus megaceros*) in 2008, and in dorcas gazelles (*Gazelle dorcus*) in 2016–2017, but the clinical, pathological, epidemiological and laboratory details related to these reports are incomplete, so there is uncertainty about the occurrence of naturally occurring PPRV disease in these wild populations [12,15]. 

Recent experimental transmission studies demonstrated that domestic pigs and European wild boar were susceptible to PPRV infection and developed clinical disease and transmitted PPRV to in-contact pigs and goats [16]. Therefore, the possible role of African and Asian wild and domestic suidae in PPR epidemiology requires further investigation.

The uncertainty around the role of wild species in PPRV transmission and maintenance is an important knowledge gap for the Global Strategy for the Control and Eradication of PPR [11,14]. It is possible that some wild species are dead-end hosts, which means that they become infected with virus through spillover from domestic animals, but there is limited or no further transmission, and therefore, they do not maintain PPRV. Evidence so far indicates that domestic cattle are likely to be dead-end hosts because, although they develop an immune response, experimental studies have not yet been able to demonstrate virus excretion or infection of in-contact susceptible cattle [17,18,19]. Alternatively, one or more wild species could be a maintenance host, or community of hosts, in which the pathogen could persist even in the complete absence of transmission from domestic hosts [20]. Few ecosystems in the world have large enough populations of wild species for the potential maintenance of PPRV in wildlife: for example, eastern Mongolia, the Boma-Gambela ecosystem in South Sudan and Ethiopia and the Greater Serengeti ecosystem in Kenya and Tanzania. Such a scenario would be a major challenge for the PPRV eradication programme. Another possible role of wild populations is that of bridge host [21], in which virus transmission is not maintained, but can persist for a while and be transmitted back (spillback) to domestic animals. In this scenario, the wild population could act as a bridge for PPRV to spread between domestic populations that are otherwise not connected. For example, during the 2016–2017 PPRV epidemic in wildlife in Mongolia, it is likely that there were multiple spillover events from livestock to wild caprines, gazelles and saiga, and an apparent subsequent spread of PPRV between wild animals, and possibly the spillback of the virus to livestock [6]. 

Considering rinderpest virus, a contagious disease of cattle that was closely related to PPRV but was finally eradicated from the world in 2011, a wide range of wild artiodactyl species was susceptible to infection, as demonstrated serologically, but clinical rinderpest disease was mainly observed in African buffalo and bovine antelopes [22,23]. In the Greater Serengeti ecosystem, outbreaks of rinderpest occurred in wildebeest, African buffalo, warthog, eland, giraffe, bushbuck and greater kudu [24,25,26,27]. Rinderpest virus would circulate for a few years in wild populations that possibly played a bridging role for infection of cattle populations, but once rinderpest virus was controlled and finally eradicated from cattle, there was no further evidence of the disease or virus circulation in wildlife populations, demonstrating that wild populations were unable to maintain virus transmission without infection in livestock [28]. Thus, no interventions were required to eliminate rinderpest virus from wildlife. Evidence so far suggests that PPRV may have a similar epidemiology in wild animals, but with a different spectrum of susceptible host species for infection and disease.

### 1.2. Greater Serengeti Ecosystem

**Figure 1:**
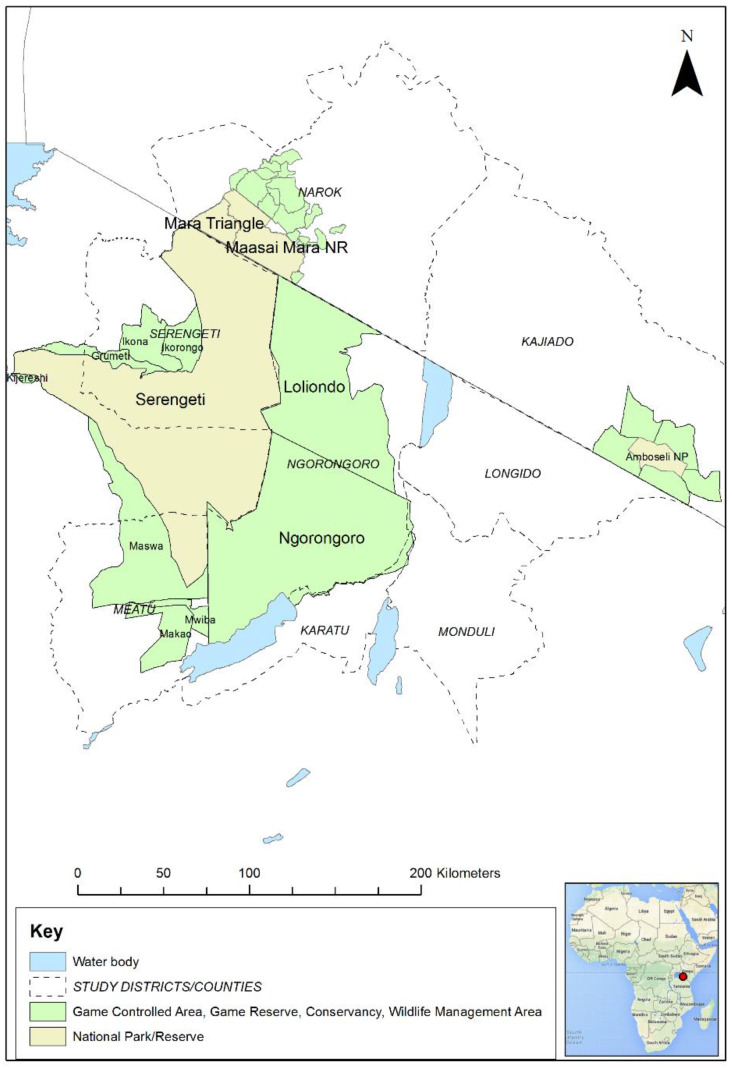
Map of the Greater Serengeti and Amboseli ecosystems showing protected areas and surrounding districts and counties in Tanzania and Kenya. Inset: map of Africa (Source: Map data © 2021 Google). The red dot indicates the location of the Greater Serengeti Ecosystem in northern Tanzania and southern Kenya.

In order to contribute to addressing this knowledge gap, field studies were carried out in the Greater Serengeti ecosystem (GSE) and Amboseli ecosystems of southern Kenya and northern Tanzania. The GSE encompasses several major protected areas: Serengeti National Park (NP), Ngorongoro Conservation Area (NCA), Loliondo Game Controlled Area (GCA) and adjoining game reserves and wildlife management areas in Tanzania, and Maasai Mara National Reserve (MMNR) and adjacent conservancies and group ranches in Kenya (Figure 1). The Amboseli ecosystem is in southern Kenya to the east of MMNR and includes Amboseli NP and nearby group ranches and wildlife sanctuaries. This study area was selected because the ecosystem supports large populations of wild artiodactyls that live in proximity to large populations of domestic sheep, goats and cattle. These domestic populations are kept mainly under transhumant pastoralist and agro-pastoralist systems, and are therefore a potential maintenance population for PPR virus. The most populous wild ungulates in the ecosystem are wildebeest (*Connochaetes taurinus*), Thomson’s and Grants gazelle (*Eudorcas thomsonii, Gazella grantii*), impala (*Aepyceros melampus*), Coke’s hartebeest (kongoni, *Alcelaphus buselaphus*), topi (*Damaliscus lunatus*), African buffalo (*Syncerus caffer*) and giraffe (*Camelopardalis giraffe*) [29]. Based on the Tanzania Wildlife Research Institute (TAWIRI) wet season aerial census of 2010, there was an estimated population of approximately 500,000 antelope and African buffalo within the Tanzanian part of the ecosystem. In the Kenyan part of the ecosystem, there were approximately 37,750 antelope and African buffalo, based on the 2014 and 2017 wet season aerial censuses [30,31]. The population of wildebeest that makes a seasonal migration within the ecosystem is estimated to number approximately 1.3 million [32]. The northern part of the GSE in Narok County, Kenya, is home to approximately 30% of Kenya’s wildlife biomass, but wildlife numbers have significantly declined in the last four decades in most parts of Kenya, including in Narok and Kajiado counties, with slower but similar trends in Tanzania, while livestock numbers have increased, especially for sheep and goats [33].

The domestic small ruminant population within and around the protected areas was estimated to be approximately 3.3 million in Longido, Monduli, Karatu, Ngorongoro, Meatu and Serengeti districts in Tanzania based on the 2007–2008 agriculture census [34], and approximately 3.6 million in Narok and Kajiado counties in Kenya in 2019 [35]. Some of the protected areas prohibit the entry of livestock, for example Serengeti NP and MMNR, although the boundaries are open so wild animals can move in and out and there are frequent incursions of livestock, usually cattle and more rarely sheep and goats, particularly during the dry season and drought when pasture and water are scarce. It is therefore likely that wild and domestic animals come into close proximity, especially in the boundary areas. Antelope range freely across these boundaries while buffalo tend to stay within the Serengeti NP and MMNR to avoid human and livestock activity. Some of the protected areas are multiple land-use systems where people, domestic animals and wild animals live in close proximity, using the same grazing and water resources, for example, NCA and Loliondo GCA in Tanzania. Wildlife tourism is a major activity and source of revenue in all these areas, and there is sport hunting in specific areas. 

The GSE is one of the very few ecosystems left on earth where domestic and wild ruminants have something of an equal status in terms of land use and population, making this a unique environment to study viral epidemiology at the interface between wild ungulates and livestock.

### 1.3. Peste des Petits Ruminants Virus in Kenya and Tanzania

PPRV is considered to be endemic in both Kenya and Tanzania. It was first officially confirmed in Kenya in 2007 in the north of the country where it caused a major epidemic [36,37,38]. By 2008, it had spread to most parts of the country, including Narok county in the south, which is home to the MMNR and adjacent conservancies and group ranches, and it was first reported in Kajiado county in 2010, in which the Amboseli ecosystem is located [39]. In 2008, PPRV was first officially confirmed in sheep and goats in Ngorongoro district in northern Tanzania, which includes NCA and Loliondo GCA and is adjacent to Serengeti NP, and then spread to other parts of northern Tanzania, although there was serological evidence of PPRV in suspected cases in small ruminants in the same area in 2004 [40,41]. Over the following two years, it was subsequently detected in the eastern, central and southern parts of the country [41,42,43,44]. Since 2008, outbreaks of PPR in sheep and goats have continued to be reported in the communities of the GSE [45,46,47].

As part of surveillance during the rinderpest eradication programmes of the Pan-African Rinderpest Campaign and the Pan-African Programme for the Control of Epizootics, serological surveillance was carried out in wildlife in East and Central Africa during 1993 to 2004 [22]. In addition to serology for rinderpest, samples were also tested for PPRV antibodies by N competitive enzyme-linked immunosorbent assay (cELISA), and all of the samples collected in Tanzania or southern Kenya were PPRV seronegative. The species sampled were African buffalo, eland (*Taurotragus oryx*), giraffe, kongoni, greater kudu (*Tragelaphus strepsiceros*), oryx (*Oryx gazella*), roan antelope (*Hippotragus equinus*), sable antelope (*Hippotragus niger*) and topi. 

Another investigation was carried out by Lembo et al. [48], in which archived sera from 243 African buffalo, 59 Thomson’s gazelle and 6 Grant’s gazelle that were collected in Serengeti NP, NCA, and Arusha and Tarangire NPs (east of Ngorongoro), and from 23 African buffalo in Katavi NP, in western Tanzania, were analysed by PPRV H cELISA (Biological Diagnostic Supplies Limited (BDSL), Irvine, UK). The samples were collected prior to 2008 and during 2008–2012 for the purposes of rinderpest surveillance, research and conservation management. None of these sera were PPRV seropositive, providing no evidence of PPRV infection in these species during this period.

Subsequently, in June 2014, a field study was carried out in NCA in which wild species that were considered likely to be susceptible to PPRV infection were purposively sampled from sites in close proximity to domestic sheep and goat flocks, to find out whether there was spillover of PPRV from domestic to wild animals [45]. Overall, 63% of the 46 sera collected from healthy wild animals were PPRV antibody positive by H cELISA. Seropositives were detected in African buffalo, Grant’s gazelle, wildebeest and impala, but not in Thomson’s gazelle. During the study, clinical disease in domestic sheep and goats within the study area was confirmed as PPR by a PPRV rapid detection test (PPRV-RDT, Peste-Test, BDSL Irvine Ltd., Irvine, UK) and PPRV reverse transcription-quantitative polymerase chain reaction (RT-qPCR). It was clear that wild animals were being exposed to PPRV; this was most likely due to spillover of infection from infected sheep and goats, but could also have been due to transmission between wild animals. No clinical disease was observed in any wild animals during this study, and all the ocular and nasal swabs collected were RT-qPCR negative, except for one sample from a Grant’s gazelle sampled within 5 km of an infected small ruminant flock. However, amplification of the PPRV genome on a gel-based PCR was not successful, so the RT-qPCR result should be interpreted with caution [45]. So far, no cases of PPR-like disease have been reported in wild species in the GSE. While a severe disease outbreak with high morbidity and/or mortality would be identified and reported by wildlife rangers and confirmed by wildlife department veterinary staff, a milder disease with low mortality could go unnoticed. There is wildlife ranger patrol-based monitoring in the Serengeti ecosystem, which includes electronic recording of observations of disease and mortality, and passive wild animal surveillance by tour drivers. These provide a relatively sensitive system to detect any significant disease events among visible wildlife, but these processes are not specifically aimed at detecting PPR disease. 

In this paper, we present the results of field studies that aimed to detect evidence of PPRV infection in a range of wild animal species in the GSE and Amboseli ecosystems, and to determine the seroprevalence of PPRV in African buffalo and Grant’s gazelle in the GSE. 

## 2. Materials and Methods

### 2.1. Purposive Sampling of Wild Animals (2015–2016)

Purposive sampling of resident and migratory wild animals (based on their known ecology in these areas) at the wildlife–livestock interface was carried out in 2015–2016 as a follow up to the 2014 NCA study of Mahapatra et al. [45]. The aim was to find evidence of PPRV infection in wild animal species in Loliondo GCA in the northern part of Ngorongoro district in Tanzania, and in the Mara and Amboseli ecosystems in Kenya. During the same period, reports of PPR-like disease in domestic sheep and goat flocks were investigated and PPRV disease was confirmed by PPRV-RDT and RT-qPCR in multiple sites in Loliondo and NCA, as described by Jones et al. [46]. Overall, 11 wild ungulate species were targeted: African buffalo, wildebeest, Grant’s gazelle, Thomson’s gazelle, impala, topi, kongoni, warthog (*Phacochoerus africanus*), waterbuck (*Kobus ellipsiprymnus*), gerenuk (*Litocranius walleri*) and lesser kudu (*Tragelaphus imberbis*). 

In Loliondo GCA, sites were identified close to the boundary of Serengeti NP, where the target species were likely to be located in areas that were also grazed by transhumant domestic small ruminant flocks, on the basis of the 2010 TAWIRI wet season aerial census. As described, African buffalo limit their range to within the National Park, so sites for African buffalo sampling were identified just inside the park boundary. In the Mara ecosystem, sites were selected in the boundaries of the MMNR and the nearby Olarra South conservancy. In the Amboseli ecosystem, sites were selected within Amboseli NP and in the neighbouring Kimana Sanctuary and Olgulului group ranch. A total of 132 animals were captured and immobilised (see details in Appendix A).

The samples collected from each animal were whole blood for serum, conjunctival and nasal swabs and faeces (see details of sample processing and storage in Appendix B). The sex and age (based on teeth eruption and wear, horn size and shape) were recorded for all animals, while the body condition score (emaciated, thin, average, good, very good), and clinical observations were recorded for animals in Tanzania only. The global positioning system (GPS) coordinates were recorded for the sampling sites. 

### 2.2. Cross-Sectional Serological Survey of the Greater Serengeti Ecosystem (2018–2019)

A cross-sectional serological survey was carried out in the GSE during 2018 and 2019, with the aim of determining the prevalence of PPRV antibody in two resident wild species that were known to be susceptible to PPRV infection based on previous studies and were accessible for capture. Resident species were targeted so that herds at varying distances from livestock populations could be sampled, to investigate the influence of the wildlife–livestock interface on exposure to PPRV. Migratory species such as wildebeest pass through multiple land-use areas and areas that exclude livestock, making it difficult to assess the effect of proximity to livestock on seroprevalence. The African buffalo was selected because of a tendency to be resident and the higher antibody prevalence detected in buffalo in earlier surveys in East Africa [13,45]. Grant’s gazelle was selected as a representative antelope that was well distributed across the ecosystem, and is relatively resident because it feeds on both grass and browse, and is therefore less dependent on migration [49]. The population of African buffalo in the GSE was estimated to be 41,500, and the population of Grant’s gazelle was 125,500 (Table 1). 

#### 2.2.1. Survey Design

**Figure 2:**
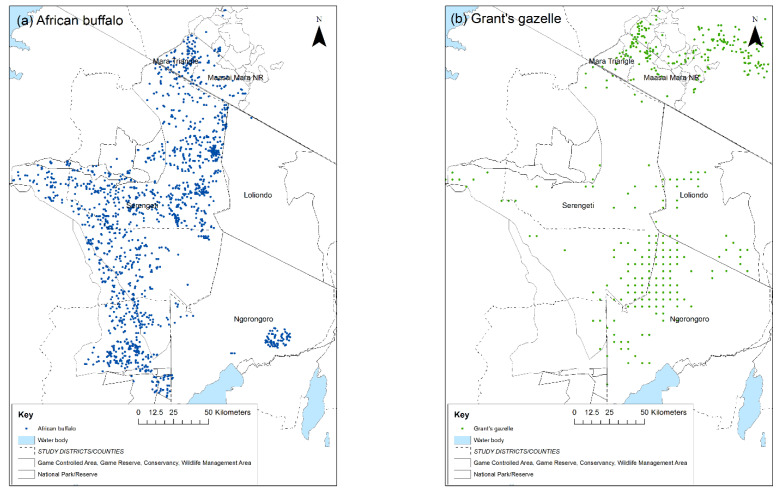
Wet season distribution of: (**a**) African buffalo; (**b**) Grant’s gazelle in the Greater Serengeti ecosystem, 2014. Source of data: Tanzania Wildlife Research Institute and Kenya Wildlife Service aerial surveys and census data. Dots represent sightings of one or more animals: blue = African buffalo, green = Grant’s gazelle.

A two-stage survey design was used with herds as the first stage and animals as the second stage. The sample size required to determine seroprevalence for a two-stage sample was estimated to be 28 sites and 5 animals per site to give a total of 140 samples from each species (90% confidence level, 10% precision, 50% expected prevalence) using a test of 94.5% sensitivity and 99.4% specificity [51]. The numbers of herds to be sampled per protected area were allocated in proportion to the population in the area (Table 1). Herds were randomly selected for sampling as follows. A total of 200 random geo-reference points across the ecosystem were generated in ArcGIS version 10 (Esri Inc http://www.esri.com, accessed on 1 March 2021). These were cross-checked against the known distribution of each species based on aerial surveys (Figure 2), and points that fell outside the species range were excluded. From the remaining points, 28 were randomly selected for each species, as well as an additional set of reserve points. The field team travelled to the selected points and searched for a herd within a 5 km radius of the point. If no animals were found, then the team moved to the nearest reserve point. On approaching some points, it was found that they were not accessible for animal capture due to rough terrain or vegetation density, in which case a reserve point was used. An area in the centre of Serengeti NP was designated as a rhino conservation area and was not accessible for animal capture. Therefore, three points falling within this area were replaced with the nearest reserve points.

In addition, a more intensive sample of African buffalo was obtained from the Mara Triangle, where the buffalo were perceived to have no or minimal contact with domestic small ruminants. The Mara Triangle forms the western part of the MMNR and is bordered to the west by the Siria escarpment, to the east by the Mara River and to the south by the international border with Tanzania. It was hypothesised that if there was little or no contact between the African buffalo in the Mara Triangle and domestic small ruminants, then exposure to PPRV would be very low and, therefore, antibody prevalence would be very low, unless there was buffalo-to-buffalo transmission of PPRV. A sample size to demonstrate the absence of PPRV antibody was calculated, sufficient to detect at least one positive animal with a 95% confidence level and 10% precision if 50% of herds had seropositive animals and the within herd seroprevalence was 20% (using a test of 94.5% sensitivity and 99.4% specificity). There were 6 herds of African buffalo within the Mara Triangle, numbering 80–350 animals per herd; therefore, all 6 herds were sampled and 8 buffalo from different age categories (<2 years, 2–3 years, 4–5 years and >6 years) were sampled from each herd, to give a total of 48 samples from this population.

#### 2.2.2. Capture and Biological Sample Collection

Once a herd of buffalo or Grant’s gazelle was identified, animals were selected for capture and immobilisation by darting or net capture to obtain a range of ages (based on horn and body size), excluding calves. The method of capture and immobilisation is detailed in Appendix A. 

A number of animals of other species were also captured and sampled during the course of the survey. Initially, a cross-sectional sample of impala was also planned, but several attempts to capture impala showed that darting this species was riskier from an anaesthetic perspective and very difficult because the flight distance was very long (at least 100 metres). Sampling of impala was therefore stopped after the first field mission, after 14 animals were sampled from 3 sites in the western part of the Serengeti NP. In addition, during net capture of Grant’s gazelle in NCA, herds of Grant’s gazelle were sometimes grazing together with Thomson’s gazelle, so some Thomson’s gazelle were captured in nets together with Grant’s gazelle. Thus, nine Thomson’s gazelle were opportunistically sampled at three sites.

From each animal, the following samples were collected: whole blood for serum, ocular and nasal swabs and faeces. For each animal, the approximate age in years based on dentition and horns [52,53,54], sex and body condition (emaciated, thin, average, good, very good) were recorded. Any clinical signs were noted, and animals were categorised as healthy (no clinical signs observed) or unhealthy (clinical signs observed). The approximate herd size and type was noted.

### 2.3. Non-Invasive Faecal Sampling of Young Wildebeest, 2018

In December 2018, a study was carried out in juvenile wildebeest to determine whether they were infected with PPRV, by the detection of PPRV ribonucleic acid (RNA) in faeces samples collected non-invasively from a purposive selection of juvenile wildebeest from the migratory wildebeest population in the GSE. This aimed to explore the hypothesis that, in a scenario in which wildebeest could maintain PPRV, the seasonal influx of immunologically naïve young individuals in this massive population would trigger a peak of PPRV infection and virus excretion in this cohort, as has been observed in other host–pathogen models such as avian influenza [55,56].

**Figure 3:**
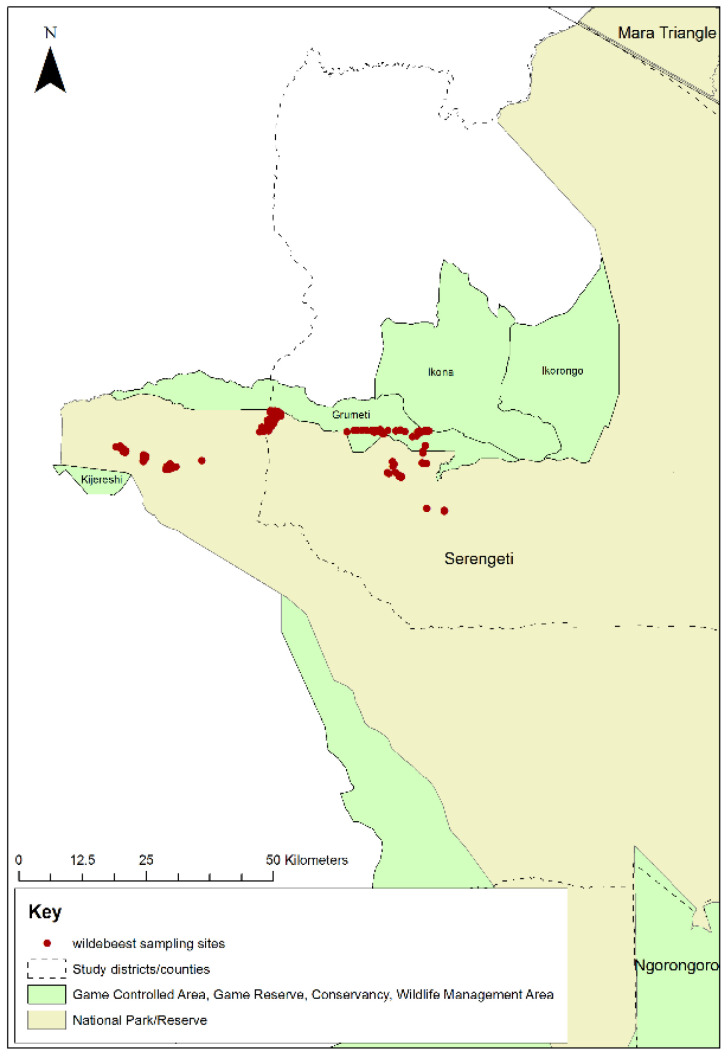
Map of Serengeti National Park showing locations where wildebeest faecal samples were collected in western Serengeti National Park and Grumeti Game Reserve, 2018.

A second aim of this study was to conduct a field trial of the non-invasive faecal sampling method for PPRV surveillance in wild animals proposed in Bataille et al. [57]. The peak time for wildebeest births was in February, therefore, after allowing at least 6 months for maternal antibody to wane, faecal samples were collected non-invasively from animals aged 8–10 months old. The annual migration of the wildebeest was tracked, and by December, the wildebeest were in the western part of the Serengeti NP. The research team travelled to this area and searched for groups of wildebeest. Upon finding a group, the team identified juvenile animals in the group and monitored them from a distance until observation of side-to-side tail movement, which indicated that an animal was defecating. The team then approached and collected a sample of the fresh faeces sufficient to fill a 5 mL cryovial. Faeces from 199 animals were collected in western Serengeti NP and the adjacent Grumeti Game Reserve in December 2018 (Figure 3).

### 2.4. Outbreak Investigations of PPR-Like Disease Reports in Domestic Sheep and Goats (2018–2019)

In order to assess whether PPRV was present in domestic small ruminants during the wildlife survey, visits were made to the livestock-keeping communities surrounding the protected areas of the GSE; the southern part of Narok County in Kenya, and Longido, Monduli, Karatu, Meatu, Ngorongoro and Serengeti districts in Tanzania. Meetings were held with the local veterinary officers to collect information on reports of PPR-like disease, and these were followed up with outbreak investigations. Suspected cases of PPR disease in sheep and goats were clinically examined. Conjunctival swabs were collected from up to five animals per flock that were showing early signs of PPR disease (pyrexia and oculo-nasal discharge) and analysed by PPRV rapid detection test (PPRV-RDT, Peste-Test, BDSL Irvine Ltd., Irvine, UK) [58]. Conjunctival and nasal swabs were collected from the same animals for PPRV RT-qPCR, whether the rapid detection test was positive or negative. Details of sample processing and storage are provided in Appendix B.

### 2.5. Laboratory Analysis

#### 2.5.1. Antibody Detection by cELISA

The serum samples were tested at the Pirbright Institute, United Kingdom (UK), for the presence of PPRV-specific antibodies. The samples collected in 2015 were analysed using an in-house anti-hemagglutinin (H) PPRV cELISA [59]. Samples with a percentage inhibition value >50 were considered to be positive. All the tests were carried out in duplicate wells and borderline positive samples were repeated to confirm results. The mean of the two results from each sample was used in subsequent analysis. The H cELISA was not available after 2015; therefore, samples collected in 2016 and in 2018–2019 were analysed using the ID Screen PPR Competition ELISA for the detection of PPRV nucleoprotein (N) antibodies following the manufacturer’s instructions (IDvet, France https://www.id-vet.com/produit/id-screen-ppr-competition/, accessed on 1 March 2021). Samples with a percentage inhibition value <50 were considered to be positive, values from 50 to 60 were considered to be doubtful, and values >60 were considered to be negative, as per the manufacturer’s instructions. It should be noted that, while both of these cELISAs were validated for use in sheep and goats, they have not yet been validated for any wild species. 

#### 2.5.2. RNA Extraction and Real-Time Reverse Transcription-Polymerase Chain Reaction (RT-qPCR)

Ocular and nasal swabs from wild and domestic animals were tested for the presence of PPRV RNA by RT-qPCR. Samples collected during the 2015–2016 study were analysed at the Pirbright Institute, while those collected during 2018–2019 were analysed at Sokoine University of Agriculture (SUA), Tanzania. A team of scientists from the Pirbright Institute and the Royal Veterinary College, UK was seconded to SUA to provide training and analyse the swab samples in RT-qPCR. Total RNA was extracted from the swab elutes manually using Qiagen RNeasy mini kit (QIAGEN GmbH, Hilden, Germany) following the manufacturer’s instructions. The presence of PPRV RNA was determined by PPRV RT-qPCR following the method of Batten et al. [60]. 

Faecal samples collected from wild animals, and from suspected cases of PPR in sheep and goats, were processed at Centre de Coopération Internationale en Recherche Agronomique pour le Développement (CIRAD), France, using the method of Bataille et al. [57] with the following modifications: high-throughput RNA extraction was carried out using the MagAttract 96 Cador Pathogen kit (Indical Bioscience, Germany) on a KingFisher Flex automated extractor (ThermoFisher, IDvet genetics, France) following the manufacturers’ instructions. The presence of PPRV RNA was determined by PPRV RT-qPCR following the method of Batten et al. [60], using the qScript XLT One-Step RT-qPCR kit (Quantabio, VWR, Fontenay-sous-Bois, France).

### 2.6. Data Analysis

For the purposive study, a descriptive analysis of the serological results was carried out to determine the overall proportion of animals that were PPRV antibody positive, and the proportions by area, species and age. The association between age in years and serological status was examined by logistic regression in Stata IC version 12.1 (StataCorp LLC, College Station, TX, USA). 

Similarly, for the cross-sectional survey, descriptive analysis of the serological results was carried out. Weighted seroprevalence estimates were calculated to take into account the variation in the number of animals sampled and herd size between sampling sites. Univariable analysis of serological status by species, country, protected area, age in years, sex and herd size was carried out for African buffalo and Grant’s gazelle, while the impala and Thomson’s gazelle results were excluded from this analysis because of the small and non-representative sample. In order to explore the effect of clustering due to the two-stage survey design, a mixed-effect logistic regression model with site as a random effect was applied. On the basis of the likelihood ratio test (*p* = 0.048), the mixed-effect model was found to be better than a logistic regression model without the random effect and was therefore used for univariable and multivariable analysis. 

To further explore the effect of age, a categorical variable was created with young, sub-adult, adult and aged categories, which were defined for buffalo as young (0.5–<1 year), sub-adult (1–<4 years), adult (4–<10 years) and aged (10 years and above), while for Grant’s gazelle this was defined as young (0.5–<1 year), sub-adult (1–<2 years), adult (2–<6 years) and aged (6 years and above). To explore the association between proximity to livestock and serological status, sites within Serengeti NP, eastern MMNR (defined as the part of the MMNR to the east of the Mara Triangle) and the Mara Triangle were categorised by their distance from the boundary (<10 km or >10 km from the boundary). All other sites in NCA and Loliondo GCA were multi-use areas and therefore categorised as <10 km from livestock.

Variables with *p* values < 0.2 were taken forward for multivariable analysis. Variables were added individually to a mixed-effect model with site as a random effect, starting with the variable with the largest odds ratio (OR) and lowest *p* value in univariable analysis. Variables were retained in the model after assessing the OR, *p* value and the likelihood ratio test comparing the model with and without the variable. 

The GPS coordinates of sampling sites were visualised in ArcGIS version 10 with publicly available shape files of Kenyan and Tanzanian administrative boundaries, and shape files of protected areas, water bodies and species distribution provided by TAWIRI and Kenya Wildlife Service (KWS). For the cross-sectional survey, the proportions of seropositive animals at each site were also displayed spatially. 

## 3. Results

### 3.1. Purposive Sampling of Wild Animals (2015–2016)

A total of 132 wild animals from 11 species were sampled from the sites in Kenya and Tanzania, of which 26 animals (19.7%) were PPRV seropositive (Table 2). All of them were categorised as healthy with body condition scores ranging from good to very good. No PPRV RNA was detected by RT-qPCR in the ocular or nasal swabs, or in the faeces collected from these animals. 

The number of animals sampled per species ranged from 1 to 34, and seropositive animals were identified in 9 of the 11 species sampled. Overall, the species with higher proportions of seropositives among the animals sampled were Grant’s gazelle, African buffalo, kongoni and Thomson’s gazelle. 

**Figure 4:**
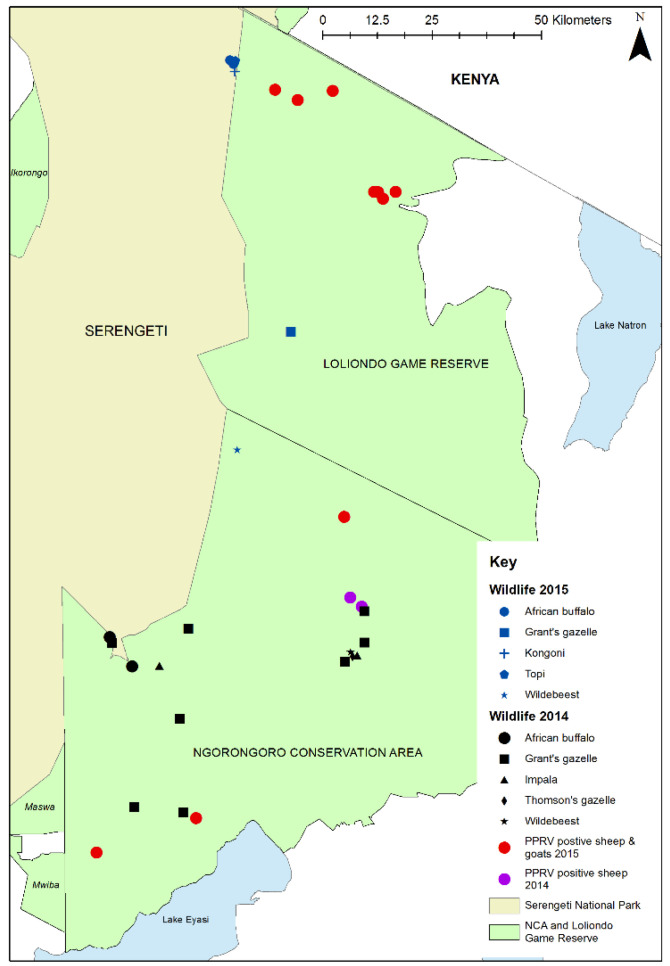
Map of Ngorongoro District showing 2015 wildlife sampling sites in Loliondo Game Controlled Area (blue symbols), 2014 wildlife sampling sites in Ngorongoro Conservation Area (black symbols) [45] and locations of confirmed PPRV in domestic flocks in 2014 (purple dots) [45] and 2015 (red dots) [46].

Focussing on the different study areas, in Loliondo GCA, Tanzania, 18 wild animals belonging to 5 species were sampled from 8 herds in 3 sites (Table 2, Figure 4), of which 11 animals (61.1%) were PPRV seropositive. High proportions of African buffalo, topi, kongoni and Grant’s gazelle were seropositive, but the juvenile wildebeest sampled from the migratory population were seronegative. Other species were not sampled because they were not found in the area at the time of sampling. 

**Figure 5:**
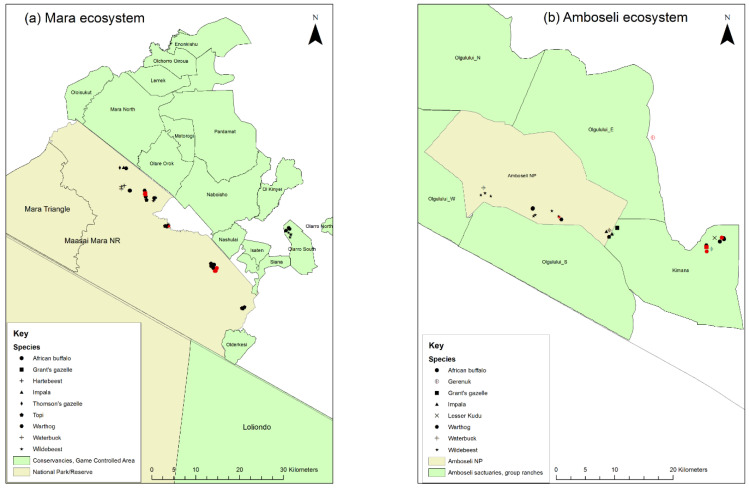
Maps of the 2016 wildlife sampling sites in Kenya: (**a**) the Mara ecosystem; (**b**) the Amboseli ecosystem. Sampling sites of seropositive animals are shown by red symbols.

In the Mara ecosystem, 57 animals of 9 species were sampled (Figure 5a) of which 8 animals (14.0%) were PPRV seropositive, all within MMNR. High proportions of seropositives were found in African buffalo, Grant’s gazelle, impala and Thomson’s gazelle, but warthog, topi, wildebeest, kongoni and waterbuck were all seronegative. 

In the Amboseli ecosystem, 57 animals from 8 species were sampled (Figure 5b) of which 7 animals were PPRV seropositive (12.3%). One positive was inside Amboseli NP (out of 30 animals, 3.3%), five positives (two warthog, one buffalo, one impala, one Grant’s gazelle) were in Kimana Sanctuary (out of 26 animals, 19.2%) and one positive gerenuk was at the boundary of Olgulului group ranch. High proportions of seropositives were found in Grant’s gazelle, while samples from waterbuck and lesser kudu were seronegative.

**Figure 6:**
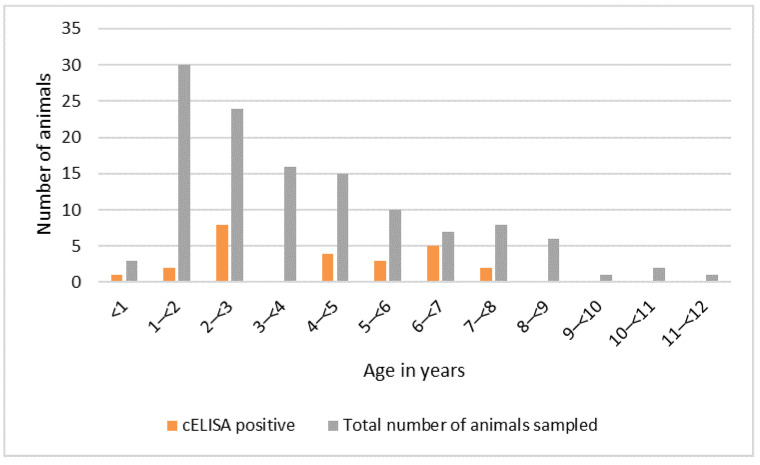
Peste des petits ruminants virus serology results by age, Kenya and Tanzania purposive wild animal sampling 2015–2016 (n = 123).

The distribution of positive sera by age of the wild animals sampled is shown in Figure 6. The ages of nine animals were not recorded (eight seronegative and one seropositive), so these were excluded from this figure. Seropositive animals were aged between six months and seven years. There was no evidence of an association between age and presence of antibody, using univariable logistic regression, although the sample size may have been too small to detect an association. There was no evidence of an association between sex and serological status. Most of the animals sampled in Tanzania were in good body condition (61%), while a few had average (22%) or very good (17%) body condition, and all were categorised as healthy. 

### 3.2. Cross-Sectional Survey in the Greater Serengeti Ecosystem (2018–2019)

During February and May 2019 in Kenya, samples were collected from 32 African buffalo in 7 herds and 5 Grant’s gazelle in 2 sites in the eastern MMNR, and 48 African buffalo from 6 herds were sampled in the Mara Triangle. Between October 2018 and August 2019 in Tanzania, samples were collected from 111 African buffalo from 22 sites and from 136 Grant’s gazelle from 27 sites in Serengeti NP, NCA and Loliondo GCA. In addition, 14 impala were sampled at three sites in western Serengeti NP, and 9 Thomson’s gazelle were sampled at two sites in NCA and one site in Loliondo. In spite of several attempts, it was not possible to sample any buffalo in the southern part of Serengeti NP because the conditions were very dry, and the few animals encountered retreated quickly to bushy areas where it was not possible to dart them. At the park boundary in this area, there is a dry season grazing and watering place for cattle, sheep and goats, so it is likely that the buffalo were avoiding the seasonal increase in human and livestock activity.

The total number of animals sampled in Kenya and Tanzania was 191 African buffalo, 141 Grant’s gazelle, 14 impala and 9 Thomson’s gazelle. A summary of the cELISA results is presented in Table 3. The weighted seroprevalence for African buffalo was 12.0% (95% CI 7.4, 18.7%), and 14.1% (95% CI 8.6, 22.2%) of buffalo were doubtful, while the weighted seroprevalence in Grant’s gazelle was 1.1% (95% CI 0.3, 4.4%), and 3.5% (95% CI 1.3, 9.1%) were doubtful (serum samples from two animals were missing). All of the impala sampled were negative, while the weighted seroprevalence in Thomson’s gazelles was 25.2% (95% CI 7.9, 56.9%), and 8.4% (95% CI 2.9, 21.9%) were doubtful. 

#### 3.2.1. RT-qPCR Results

For the wild animals sampled in Tanzania, ocular, nasal or oral swabs from 269 out of 270 animals were screened for PPRV RNA by RT-qPCR (samples were missing for one Grant’s gazelle), and all were negative indicating the absence of active virus circulation among these animals at the time of sampling (Table 4). Unfortunately, it has not yet been possible to screen the swabs collected from the wild animals sampled in Kenya. However, faecal samples from 340 wild animals in both Tanzania and Kenya were screened for PPRV RNA by RT-qPCR, and all were RT-qPCR negative. 

There were no clinical signs of PPR disease among the wild animals sampled. Most were in very good (67.2%) or good (27.1%) body condition, while 5.4% were in average condition, and one animal was in poor condition (0.3%). Only five animals had other observable clinical signs (skin lesions, hair loss, swollen lymph nodes or swollen joints): four of these were seronegative and one had a doubtful result.

#### 3.2.2. Analysis of Serology Results

**Figure 7:**
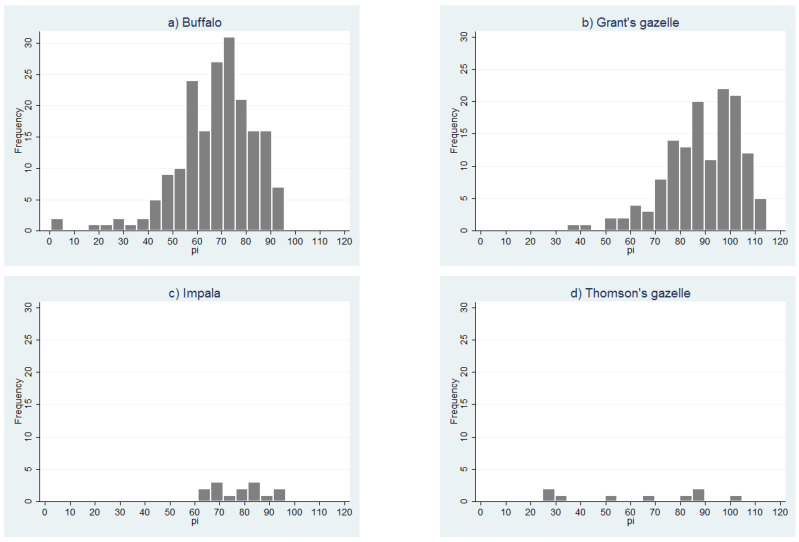
Distribution of peste des petits ruminants virus competitive enzyme-linked immune scheme 2018. (**a**) African buffalo; (**b**) Grant’s gazelle; (**c**) impala; (**d**) Thomson’s gazelle.

Given that the PPRV cELISA has not yet been validated for any of the wild species sampled in this study, the distribution of the PI values was examined for each species (Figure 7a–d). The number of samples for impala and Thomson’s gazelle was too small to discern any pattern, but the histograms for African buffalo and Grant’s gazelle showed a left-skewed distribution, with no obvious separation between positive and negative populations. For the purposes of analysis, all doubtful results were considered to be seronegative (interpretation 1), but in order to explore the effect of varying the positive cut-off for the cELISA, a second analysis was carried out in which all doubtful results were considered to be seropositive (interpretation 2, results presented in Table S1). 

**Figure 8:**
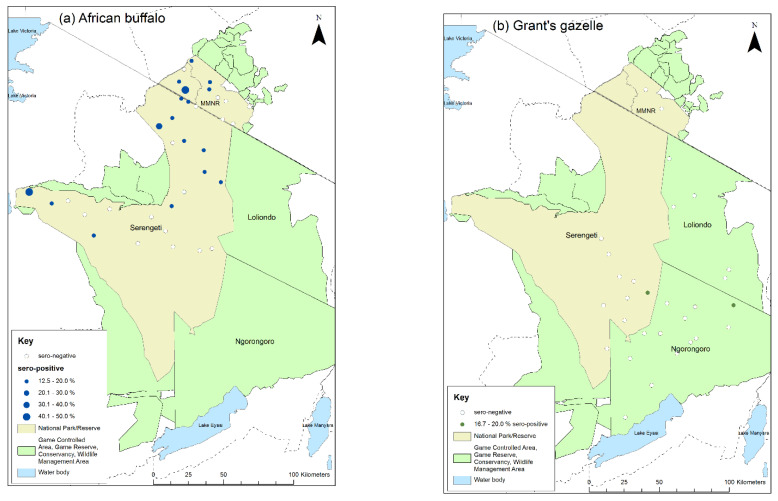
Map of sampling sites for the cross-sectional survey 2018–2019 indicating the proportion of cELISA positive animals at each site: (**a**) African buffalo; (**b**) Grant’s gazelle. Positive animals are those with percentage inhibition <50 (interpretation 1).

The spatial distribution of the sampling sites for African buffalo and Grant’s gazelle, and the proportion of animals that were seropositive at each site is shown in Figure 8 for interpretation 1, and in Figure S1 for interpretation 2. African buffalo were sampled at 35 sites with a median of five animals per site (range 1–8) (Figure S2). As planned, eight animals were sampled at each of the six herds in the Mara Triangle, and five animals were sampled at 24 sites in the other parts of the ecosystem. However, in the remaining sites only one animal was sampled at one site, four animals were sampled at another, and six animals were sampled at three sites. Positive animals were detected at 17 sites (48.6%) for interpretation 1 and 28 sites (89.0%) for interpretation 2.

Grant’s gazelles were sampled at 29 sites with a median of five animals per site (range 1–9) (Figure S2). There was more variation in the number of gazelle sampled per site compared with buffalo. Five animals were sampled as planned at 11 sites, while four animals were sampled at 6 sites, six animals at 4 sites, and three animals at 3 sites. For the remaining five sites, one, two, seven, eight and nine animals were sampled. Positive animals were detected at two sites (6.9%) by interpretation 1, one in Serengeti NP and one in NCA, and at six sites (20.7%) by interpretation 2, four in Serengeti NP and two in NCA (Figure 8, Figure S1).

In most sites, animals were sampled from a single herd, but in a few sites, animals were sampled from more than one herd in order to achieve the required sample size per site. For the African buffalo, 40 herds were sampled at the 35 sites with a median herd size of 100 (range 2 to 900). For the Grant’s gazelle, 33 herds were sampled at the 29 sites, with a median herd size of 13 (range 2–60). For impala, 10 herds were sampled at three sites, with a median herd size of 23 (range 6–50). For Thomson’s gazelle, one herd was sampled at each of the three sites, with a median herd size of 16 (range 12–40).

#### 3.2.3. Univariable and Multivariable Analysis

The unweighted proportions of African buffalo and Grant’s gazelle that were seropositive by species, country, protected area, age (in years), age category, sex, herd size and proximity to livestock are shown in Table 5 (interpretation 1). The results when interpretation 2 was applied are in Table S1.

For interpretation 1, there was no evidence of an association between serological status and country, protected area, age category, sex or proximity to livestock. However, there was a strong association between serological status and species—an African buffalo had nine times the odds of being seropositive than a Grant’s gazelle (OR 9.5, 95% confidence interval (CI) 2.1, 42.6, *p* = 0.003). There was also a strong association between serological status and age in years—for each one-year increase in age there was a 20% increase in odds of being positive (OR 1.2, 95% CI 1.0, 1.4, *p* = 0.0011). The mean herd size for positive animals was 201.5 (standard deviation (SD) 210.7), while for negative animals it was 118.6 (SD 164.6). There was weak evidence of an association between herd size and serological status—for an increase in herd size of one animal there was a 1.002 increase in odds of being positive (95% CI 1.00, 1.01, *p* = 0.048). Three variables—species, age in years and herd size—were taken forward for multivariable analysis. Starting with the mixed-effect model containing species with site as a random effect, neither age in years nor herd size improved the model; therefore, the final model was the univariable model containing species, as shown in Table 5. After adjusting for species, there was no longer an effect of age or herd size. This was because buffalo were more likely to be positive than Grant’s gazelle, and the average age of buffalo (6 years) was higher than for Grant’s gazelle (3 years), while the average herd size for buffalo (202, SD 189) was higher than for Grant’s gazelle (20, SD 13).

Similarly, for interpretation 2, in univariable analysis there was some evidence of an association between serological status and species, age in years and herd size, as well as country and protected area, but in a mixed-effect model containing species with site as a random effect, none of these variables improved the model (Table S2).

### 3.3. Results of Non-Invasive Sampling of Wildebeest Faeces Study, 2018

All the 199 immature wildebeest that were sampled were apparently healthy, apart from 1 animal that was observed to be weak. The consistency of the faeces in most animals was considered to be normal (faecal pellets), but 18 animals (9.1%) had abnormal faeces, either liquid (1 animal), faeces containing mucus (8 animals) or faeces containing mucus and blood (9 animals). All the 199 wildebeest faeces samples were negative for PPRV RNA by RT-qPCR.

### 3.4. Outbreak Investigations of PPR-Like Disease Reports in Domestic Sheep and Goats 2018

**Figure 9:**
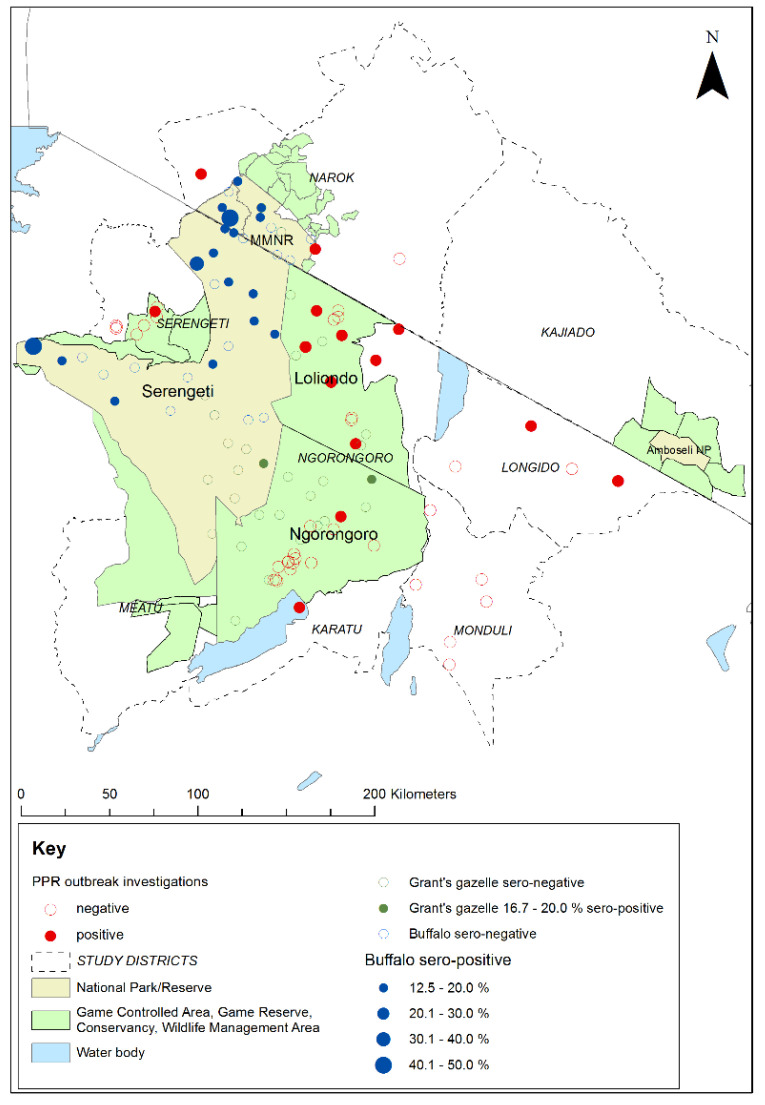
Map of the Greater Serengeti and Amboseli ecosystems showing locations of peste des petits ruminants virus (PPRV) positive and negative investigations of PPR-like disease reports in sheep and goat flocks, in relation to the 2018–2019 cross-sectional survey sampling sites and PPRV seroprevalence for African buffalo and Grant’s gazelle. Positive disease investigations were defined as at least one animal positive by PPRV rapid detection test and/or real-time reverse transcription-polymerase chain reaction.

During the study period, 64 flock investigations of PPR-like disease reports were carried out in the six study districts in Tanzania, and one in Narok County in Kenya (Table 6). The locations and results of the investigations are shown in Figure 9.

Among the Tanzanian investigations, samples were collected for PPRV-RDT from 88 animals in 38 flocks, and swabs were collected for RT-qPCR from 54 animals in 25 flocks. Overall, 20 animals from 16 flocks were confirmed to be infected with PPRV by rapid detection test and/or RT-qPCR. These positive flocks were in 12 wards in 4 districts. Very few disease reports were received in the Kenyan part of the study area, and only one outbreak investigation was carried out in a village near to Morijo in Narok South, during which two PPRV RDT tests were carried out and both were negative. However, during a PPRV vaccination campaign carried out by the Narok County veterinary services during June 2019, the vaccination team identified suspected cases of PPRV disease in several flocks at Oloolaimutia, close to the eastern boundary of MMNR, and at Lolgorian, in Kilgoris sub-county, approximately 15 km north of the Mara Triangle. Using the PPRV rapid detection test, PPRV was detected in flocks at both locations: at Oloolaimutia, 1 out of 23 sheep and goats tested was positive, and at Lolgorian, 9 out of 40 sheep and goats were positive. The results of these investigations in Kenya and Tanzania demonstrate that PPRV was circulating in the study area at the time of the cross-sectional wildlife survey. 

## 4. Discussion

The results of the purposive sampling of wild animals in 2015–2016 provide further evidence of the natural exposure to PPRV infection in a range of wild artiodactyl species living in proximity to domestic small ruminants. Among these were six Bovidae species that were previously found to be seropositive: African buffalo, Grant’s gazelle, Thomson’s gazelle, topi, impala and wildebeest. In addition, as far as the authors are aware, this is the first time that evidence of natural infection has been demonstrated in two other Bovidae species, kongoni and gerenuk. Seropositives were also found in warthog from the Suidae family. These findings complement and add to the results of the 2014 purposive study in NCA in which African buffalo, Grant’s gazelle, wildebeest and impala were found to be seropositive [45]. The detection of active PPRV infection in domestic small ruminants at multiple sites in Loliondo GCA and NCA at the time of the purposive study [46] suggests that a likely source of infection for the wild artiodactyls was infected sheep and goats with which they share resources, although transmission of PPRV within or between wild species cannot be ruled out. The small number of purposively selected animals that were sampled per species means that the observed proportion of seropositive animals per species, or absence of antibody, is unlikely to be representative of the wider population, and is therefore not generalizable. In addition, presence of PPRV antibody indicates exposure to PPRV infection at some time in the past rather than current infection, and therefore it is not possible to determine whether the seropositive animals were exposed to infection simultaneously, or individuals were exposed at different times. An increase in seroprevalence with age could indicate cumulative exposure over time, but this pattern was not evident in this purposive dataset, although the sample size was probably too small to detect an association with age. 

Previous PPR serological surveys in wild animals conducted during rinderpest surveillance activities in East and Central Africa (1994–2004) found small numbers of seropositive African buffalo, topi, warthog and eland in southern Ethiopia [22], but no seropositives among kongoni, wildebeest, Grant’s gazelle or impala, although the number of samples collected for these species was small [61]. A larger number of samples were collected from African buffalo and seropositives were found in both West Africa [62] and East Africa [22,61]. Among a purposive sample of African buffalo and Ugandan kob (*Kobus kob thomasi*) collected in 2015 and 2017 in Queen Elizabeth NP in western Uganda, 19.0% of buffalo and 10.3% of Ugandan kob were seropositive [13]. In the Serengeti ecosystem, samples collected from wild animals during 2008 to 2012 were all seronegative [48]. During this period, PPRV was first officially confirmed in sheep and goats in Ngorongoro district and spread across the north of Tanzania to small ruminant populations in the east, centre and south of the country. The species sampled were Thomson’s gazelle in NCA and Serengeti NP, Grant’s gazelle in NCA, and African buffalo in Serengeti NP and the Ngorongoro crater in MCA [48]. However, a few years later in 2014, Mahapatra et al. [45] detected seropositive African buffalo, Grant’s gazelle, wildebeest and impala in NCA in herds sharing resources with domestic sheep and goat flocks in which PPR disease was confirmed to be circulating, suggesting possible spillover of PPRV from domestic to wild animals.

In order to obtain a more representative sample from wild animals in the Greater Serengeti ecosystem, the cross-sectional survey was designed using GPS coordinates to randomly select herds of African buffalo and Grant’s gazelle. To the authors’ knowledge, this is the first time that a large-scale randomised survey has been carried out in wild animals to investigate exposure to PPRV. The method could be replicated in the future to monitor the PPRV seroprevalence in these two species over time in relation to the occurrence of PPRV in domestic flocks and efforts to eliminate PPRV through vaccination. If PPRV can be maintained by wild animals in this ecosystem, then the level of seroprevalence will be maintained even if PPRV is eliminated from sheep and goats in the region. Conversely, if wild animals cannot maintain PPRV in this ecosystem then, if PPRV is eliminated from the sheep and goats, the seroprevalence in wild animals will decrease overtime to zero because of population turnover and an increasing proportion of younger naïve animals as the incidence in livestock decreases to zero.

The results of the 2018–2019 serological survey using the standard cELISA interpretation showed that there was a relatively low seroprevalence in African buffalo (12.0%, 95% CI 7.4, 18.7%), with one or more positive animals in approximately half of the sampled herds, and a very low prevalence in Grant’s gazelle (1.1%, 95% CI 0.3, 4.4%), in comparison with the results of the 2015–2016 purposive sampling focusing on the wildlife–livestock interface, in which 41.7% African buffalo and 71.4% Grant’s gazelle were seropositive. An important limitation for PPRV serological studies in wild animals is the fact that the PPRV H and N cELISAs have not been validated for use in these species. In this study, we applied the goat/cattle cut-off as determined by Libeau et al. [51], which gives a sensitivity of 94.5% and a specificity of 99.4% for this assay. In order to explore the effect of varying the cut-off, the results were also analysed with a higher cut-off by considering doubtful results as positives. Previous studies suggested that the H cELISA has a lower sensitivity in cattle compared to sheep and goats [63]. Discrepancies between PPRV H cELISA and neutralisation tests in buffalo sera have also been reported, highlighting the possibility that differential antiviral immune responses among host species may affect serological results and interpretation [64]. Further research is in progress to analyse these serum samples by a virus neutralisation test and several new serological tests, in order to inform the interpretation of the N cELISA in these species. 

PPRV seroprevalence in sheep and goats in endemic areas typically ranges from 30% to 70%, as shown by surveys in lowland Ethiopia [65], southern Tanzania [66], northern Kenya [67], northeast Uganda [68] and Sudan [69]. A serological survey in sheep and goats in northern Tanzania in 2008–2009, found a PPRV seroprevalence of 52.8% in Arusha region (which includes the eastern part of the GSE), 43.0% in Kilimanjaro region and 27.3% in Manyara region [41]. A more recent survey in the same region found a seroprevalence of 27.6% in sheep and goats compared to 11.3% in cattle, but the seroprevalence in all species was higher in pastoral villages compared to agro-pastoral villages [63]. Abubakar et al. [18] conducted a survey in a PPRV endemic area of Pakistan and found a seroprevalence of 10.0% in cattle and 14.6% in domestic water buffalo (*Bubalus bubalis*), with increasing seroprevalence with age. The seroprevalence in African buffalo found in this study is therefore similar to that found in domestic cattle and buffalo in areas of endemic PPRV in sheep and goats.

It is generally accepted that PPRV antibodies persist for life in sheep and goats after PPRV infection [70], and it is likely that this also applies to wild species. Given that African buffalo have a longer average lifespan than sheep and goats, the relatively low seroprevalence in African buffalo suggested that sustained transmission of PPRV in this species was unlikely, because there would be a cumulative exposure to PPRV over time, leading to higher seroprevalence with age, as has been reported for cattle, sheep and goats [71]. Exposure to PPRV infection was more likely to be due to sporadic spillover from domestic sheep and goats, with or without some subsequent transmission between wild animals. PPR disease is actively circulating in domestic sheep and goats in the ecosystem, as documented in previous studies [45,46,47] and demonstrated by the confirmation of PPR disease at multiple sites during this study. The very low prevalence in Grant’s gazelle, which are mainly present in areas frequented by sheep and goat flocks, suggests that virus spillover is a rare event for this species, and there is limited or no intra-species transmission. 

The relatively higher seroprevalence in African buffalo compared to Grant’s gazelle, may be due to larger herd sizes and tendency for close aggregation and residency, with a higher probability of groups coming into contact with infected pasture or water sources at key livestock interface points. African buffalo were highly susceptible to rinderpest virus infection and disease [72], but, although they are susceptible to PPRV infection, it is apparently a subclinical infection, suggesting low viral loads and low virus excretion. However, it is possible that under greater stress, as more pressure is put on wildlife systems in Africa, PPRV could be clinically expressed, as has been seen in African wild species kept under semi-managed conditions in the Middle East [8,73,74] and in wild populations in Asia [6]. 

There was no evidence of an association between serological status and proximity to livestock (defined as location within a mixed land-use area or within 10 km of a national park/reserve boundary) with seropositive buffalo detected in multiple herds both near to and further from the boundaries of Serengeti NP and MMNR. Given that this is a resident species, exposure to PPRV could have been due to indirect transmission from infected sheep and goats at shared resources or when passing through the protected area for trade or migration. Alternatively, there could be virus transmission between buffalo herds or between another wild species and buffalo, raising the possibility that African buffalo, with or without other wild species, could act as bridge hosts in this ecosystem. However, there were limitations in the use of distance from the boundary as a method of determining proximity to livestock. The borders of Serengeti NP and MMNR are open, and therefore, wild and domestic animals can potentially move freely in and out. Sheep and goats are known to enter the national parks and reserves, but the frequency of incursion is unknown, and the range of the African buffalo and Grant’s gazelle was not determined. Therefore, some animals could have been misclassified with respect to their proximity to livestock. 

Prior to the study, it was believed that there was little or no contact between wild animals within the Mara Triangle and domestic livestock, but during the study, it was discovered that there was some sharing of resources by wild and domestic animals. Personnel from the Mara Conservancy, the organisation that manages the Mara Triangle, reported that cattle, sheep and goats belonging to the community on the Siria escarpment adjacent to the Mara Triangle were allowed to enter twice per month to access a salt lick that was also used by wild animals. In addition, during interviews with livestock keepers living on the escarpment, it was reported that both wild and domestic animals grazed on the slopes of the escarpment and that wild animals visited the domestic animal feed troughs that were used to provide salt for sheep and goats. Therefore, the degree of wild-domestic animal contact was likely to be similar for the Mara Triangle as for the rest of the MMNR and the border areas of the Serengeti NP. During the dry season and periods of drought, livestock are taken into the national park or reserve to graze at night, particularly in the west of Serengeti NP, although this is mostly cattle rather than sheep and goats. Behavioural studies in southern Africa found that African buffalo tended to avoid the presence of cattle, with limited overlap of resource use in the wet season, but increasing overlap in the dry season around water sources [75,76]. In 2018, line transects were carried out in three areas bordering the MMNR to observe the grazing proximity of livestock and PPRV susceptible wild animals, during which there were seven observations of sheep and/or goats in close proximity to Thomson’s gazelle and/or impala, including one observation of sheep grazing inside the MMNR close to wildlife [77].

During the outbreak investigations in domestic small ruminants, the livestock keepers said that they regularly saw several species of wild herbivore in the grazing areas used by their flocks, but they did not observe any disease in the wild animals and they did not associate the disease in their flocks with wild animals. Thomson’s gazelle, Grant’s gazelle, wildebeest and impala were seen in all areas, while buffalo were mentioned in Ngorongoro district (NCA and Loliondo GCA). The livestock keepers reported that they did not see direct physical contact between wild animals and livestock, but the wild and domestic animals came as close as 10 m to each other and moved across the same areas of grazing land within a few minutes to a few hours. Given that in semi-arid and arid conditions, aerosol spread of PPRV is likely to require close association within a few metres, it is more likely that transmission between domestic and wild animals occurs indirectly via pasture, water or salt bodies. The conditions under which indirect transmission can occur requires investigation.

The sample size of the cross-sectional survey was designed to determine the PPRV seroprevalence, but the opportunity was taken to collect ocular and nasal swabs and faecal samples to conduct PPRV RT-qPCR. All the samples were negative for PPRV RNA; however, given that this was a random sample of mostly healthy animals, there was a low probability that any would be shedding PPRV. If an infected animal were able to shed the virus, it would do so for approximately 10 days, assuming similar dynamics to PPRV-infected sheep and goats [78,79]. Similarly, all the faecal samples collected from young wildebeest were PPRV RNA negative. To increase the likelihood of detecting PPRV in wild animals, active surveillance for clinical cases showing any of the PPR clinical signs should be carried out, and samples should be collected from sick animals. Clinical PPR disease has so far only been confirmed in Africa in captive Dorcas gazelle in Sudan and possibly free-ranging wildlife in Sudan [15], but these reports remain clinically and epidemiologically unconfirmed. However, clinical disease and PPRV RNA have been found in African species of wild artiodactyls kept under semi-managed conditions in the Middle East, including impala, Thomson’s gazelle, Dorcas gazelle, springbok (*Antidorcas marsupialis*), bushbuck (*Tragelaphus scriptus),* Nubian ibex *(Capra ibex nubiana)* and gemsbok *(Oryx gazelle)* [8,73,74], so it is possible PPR disease does occur in wild species in the GSE, but under these ecological conditions, the signs could be relatively mild or non-specific and therefore not detected or reported. 

In order to explore the possible role of other wild species in PPRV transmission, future studies in the GSE might focus on wildebeest, impala or Thomson’s gazelle. The opportunistic net capture of Thomson’s gazelle during this study indicates that this method might be feasible for the capture of an adequate sample size for this species, and non-invasive sampling techniques can be applied for species that are difficult to capture in adequate numbers. It is possible that a multi-species population could maintain PPRV through transmission within and between species, with different species playing different roles. Some species might have higher effective transmission rates, while others have lower rates, and some are unable to transmit the virus (dead-end hosts). If the overall population size of species with higher transmission rates is large, it might be sufficient to maintain virus transmission without burnout. The presence of dead-end hosts could have a dilution effect if they make up a relatively high proportion of the population. During experimental infection of sheep and goats, virus excretion has been demonstrated for animals with limited clinical signs [80,81], and it is likely that under field conditions, animals with very mild clinical signs play a role in virus transmission and maintenance. Transmission by sub-clinically infected dogs has been demonstrated for canine distemper virus, another morbillivirus [82]. Therefore, it is possible that some wild species could develop mild clinical signs and transmit a virus and, if there is an adequate supply of naïve animals to be infected, they could play a role as a bridge or maintenance host. Experimental infection and transmission studies with wild species would demonstrate whether intraspecies transmission occurs, and mathematical modelling of wild animal populations would indicate whether a virus could be maintained in those species that are able to transmit virus.

## 5. Conclusions

These studies provide serological evidence of PPRV infection in a range of wild artiodactyl species at the wildlife–livestock interface in the Greater Serengeti and Amboseli ecosystems. The cross-sectional survey found that the PPRV seroprevalence was relatively low in African buffalo and very low in Grant’s gazelle, in comparison to purposive studies in these species and surveys in domestic small ruminants. These findings suggest that exposure to PPRV infection in these species under these ecological conditions is likely to be via sporadic spillover from infected sheep and goats with possible limited transmission within wild species, and that sustained transmission in these two wild species is unlikely. Further studies are required of other major species in this ecosystem, such as impala, Thomson’s gazelle and wildebeest, in other major ecosystems with large multi-species population, and in wild populations in which confirmed PPR clinical disease occurs. The cross-sectional serological survey provides a replicable method for representative sampling of a wild animal population, which will be valuable for the future monitoring of seroprevalence in wild animals in this ecosystem. If PPRV is eliminated from the sheep and goat population in this region through livestock vaccination, the effect on seroprevalence in wild animals will indicate whether this multi-species system can maintain PPRV transmission in the absence of disease in livestock.

## Figures and Tables

**Table 1:** Estimated populations of African buffalo and Grant’s gazelle in the Greater Serengeti ecosystem and planned sample size.

Country	Area	African Buffalo Estimated Population	Grant’s Gazelle Estimated Population	African Buffalo Sample Size	Grant’s Gazelle Sample Size
Kenya	Mara Ecosystem—Maasai Mara National Reserve, group ranches, community and private conservancies	9466	5797 (3478 in MMNR)	6 herds × 5 animals = 30	1 herd × 5 animals = 5
Tanzania	Serengeti National Park	32,000	35,688	22 herds × 5 animals = 110	8 herds × 5 animals = 40
	Ngorongoro Conservation Area	-	62,280	-	14 herds × 5 animals = 70
	Loliondo Game Controlled Area	-	20,829	-	5 herds × 5 animals = 25
	Maswa Game Reserve	-	345	-	-
Total		41,446	125,504	28 herds × 5 animals = 140 animals	28 herds × 5 animals = 140 animals

Source of population data: 2014 wet season aerial census, Mara ecosystem, Kenya [31]; 2017 wet season aerial census, Mara ecosystem, Kenya [30]; 2010 wet season aerial census report and 2009 buffalo census, Tanzania [50].

**Table 2:** Serology results of purposive wild animal sampling, Tanzania and Kenya, 2015–2016.

Species	Loliondo GCA 2015		Mara Ecosystem 2016		Amboseli Ecosystem 2016		Total	
	No. Sampled	No. Seropositive (%)	No. Sampled	No. Seropositive (%)	No. Sampled	No. Seropositive (%)	No. Sampled	No. Seropositive (%)
African buffalo	7	6 (85.71)	10	3 (30.00)	7	1 (14.29)	24	10 (41.67)
Wildebeest	4	0 (0)	9	0 (0)	8	1 (12.28)	21	1 (4.76)
Topi	2	1 (50.00)	11	0 (0)	0	-	13	1 (7.69)
Kongoni	3	2 (66.67)	2	0 (0)	0	-	5	2 (40.00)
Grant’s gazelle	2	2 (100.00)	2	2 (100.00)	3	1 (33.33)	7	5 (71.43)
Impala	0	-	4	2 (50.00)	13	1 (7.69)	17	3 (17.65)
Thomson’s gazelle	0	-	3	1 (33.33)	0	-	3	1 (33.33)
Warthog	0	-	15	0 (0)	19	2 (10.53)	34	2 (5.88)
Waterbuck	0	-	1	0 (0)	5	0 (0)	6	0 (0)
Gerenuk	0	-	0	-	1	1 (100.00)	1	1 (100.00)
Lesser kudu	0	-	0	-	1	0 (0)	1	0 (0)
Total	18	11 (61.11)	57	8 (14.04)	57	7 (12.28)	132	26 (19.70)

Note: in each area, some species were not sampled because they were not present at the sampling sites or it was not possible to capture them within the timeframe of the fieldwork.

**Table 3:** Results of peste des petits ruminants virus (PPRV) competitive enzyme-linked immunosorbent assay (cELISA) of wild animal serum samples by species during the cross-sectional survey in the Greater Serengeti ecosystem, 2018–2019.

Species		Positive (PI < 50)		Doubtful (PI 50–60)		Negative (PI > 60)	
	No. Serum Samples Tested	No.	Weighted * Percentage (95% CI)	No.	Weighted Percentage (95% CI)	No.	Weighted Percentage (95% CI)
African buffalo	191	23	11.96	26	14.06	142	73.98
			(7.44, 18.66)		(8.57, 22.22)		(65.93, 80.69)
Grant’s gazelle	139	2	1.07	4	3.48	133	95.46
			(0.25, 4.43)		(1.28, 9.12)		(90.16, 97.97)
Impala	14	0	0 (0)	0	0 (0)	14	100.00
Thomson’s gazelle	9	3	25.21	1	8.40	5	66.39
			(7.92, 56.90)		(2.92, 21.86)		(29.79, 90.19)
Total	353	28	10.99	31	12.89	294	76.13
			(6.80, 17.26)		(7.87, 20.41)		(68.24, 82.56)

* Weighted seroprevalence estimates were calculated to take into account variation in the sampling fraction between sampling sites. Abbreviations: confidence interval (CI), percentage inhibition (PI).

**Table 4:** Results of peste des petits ruminants virus (PPRV) real-time reverse transcription-polymerase chain reaction in wild animal samples by species during the cross-sectional survey in the Greater Serengeti ecosystem, 2018–2019.

Species	Ocular, Nasal or Oral Swabs		Faecal Samples	
	No. Tested	No. Positive	No. Tested	No. Positive
African buffalo	111	0	185	0
Grant’s gazelle	135	0	133	0
Impala	14	0	14	0
Thomson’s gazelle	9	0	8	0
Total	269	0	340	0

**Table 5:** Peste des petits ruminants virus antibody competitive enzyme-linked immunosorbent assay results for 191 African buffalo and 139 Grant’s gazelle (percentage inhibition <50 = positive) and univariable analysis (mixed-effect logistic regression with site as random effect) (n = 330).

Variable	Category	Number Sampled	Number Positive (*%, 95% CI)	Odds Ratio (95% CI) Wald Test *p* Value
Species	African buffalo	191	23 (12.04, 7.40–16.69)	9.52 (2.13–42.57) *p* = 0.003
	Grant’s gazelle	139	2 (1.44, −0.55–3.43)	reference
Country	Kenya	85	10 (11.76, 4.85–18.68)	2.07 (0.73–5.85) *p* = 0.17
	Tanzania	245	15 (6.12, 3.10–9.14)	reference
Protected area	Eastern MMNR	37	2 (5.41, 2.01–12.82)	0.55 (0.11–2.86) *p* = 0.483
	Mara Triangle	48	8 (16.67, 5.97–27.36)	2.05 (0.68–6.13) *p* = 0.201
	Serengeti NP	154	14 (9.09, 4.52–13.66)	Reference
	NCA	67	1 (1.49, 1.44–4.43)	0.15 (0.18–1.20) *p* = 0.073
	Loliondo	24	0 (0)	-
Age**	0.5–<1	6	0 (0)	1.19 per 1 year increase (1.04–1.37) *p* = 0.011
	1–<2	35	0 (0)	
	2–<3	41	3 (7.32)	
	3–<4	65	3 (4.62)	
	4–<5	46	0 (0)	
	5–<6	21	1 (4.76)	
	6–<7	29	6 (20.69)	
	7–<8	17	3 (17.65)	
	8–<9	25	4 (16.00)	
	9–<10	5	1 (20.00)	
	10–<11	33	3 (9.09)	
	11–<12	2	1 (50.00)	
	12–<13	1	0 (0)	
	13–<14	1	0 (0)	
	14+	3	0 (0)	
Age category	Young	6	0 (0)	-
	Sub-adult	70	3 (4.29, −0.52–9.08)	0.52 (0.14–1.90) *p* = 0.319
	Adult	213	17 (7.98, 4.32–11.64)	Reference
	aged	41	5 (12.20, 2.02–22.37)	1.50 (0.47–4.72) *p* = 0.491
Sex	Female	168	10 (5.95, 2.35–9.55)	Reference
	Male	162	15 (9.26, 4.77–13.75)	1.51 (0.62–3.67) *p* = 0.368
Herd size	Range 1–900	Median 50	Mean herd size if seropositive 201.84 (210.68), if seronegative 118.56 (SD 164.59) *t*-test *p* value 0.018	1.002 per 1 animal increase (1.000–1.005) *p* = 0.048
Proximity to livestock	<10 km	217	14 (6.45, 3.16–9.74)	0.76 (0.28–2.11) *p* = 0.602
	>10 km	113	11 (9.73, 4.22–15.24)	reference

* unweighted seroprevalence estimates are reported in this table. Clustering by sample site is accounted for in the analysis by inclusion of sampling site as a random effect in the model. ** mean age if seropositive 6.36 years (SD 2.69), if seronegative 4.60 years (SD 3.00), *t*-test *p* value = 0.0048. Abbreviations: confidence interval (CI), standard deviation (SD), Maasai Mara National Reserve (MMNR), national park (NP), Ngorongoro Conservation Area (NCA).

**Table 6:** Investigation of peste des petits ruminants (PPR)-like disease in sheep and goat flocks in the study area during Feburary 2018 to July 2019.

District or County	No. Outbreak Investigations (No. Wards)	No. Investigations Sampled	No. Investigations PPRV Positive * (No. Wards)
Karatu	1 (1)	1	1 (1)
Longido	7 (4)	6	3 (2)
Monduli	11 (4)	7	0
Serengeti	10 (5)	8	1 (1)
Ngorongoro (NCA)	17 (6)	14	1 (1)
Ngorongoro (Loliondo and Sale divisions)	18 (7)	18	10 (7)
Meatu	0 (0)	-	-
Total Tanzania	64 (27)	54	16 (12)
Narok, Kenya	1 (1)	1	0 (0)

* at least one animal was positive by peste des petits ruminants virus (PPRV) rapid detection test and/or PPRV real-time reverse transcription-polymerase chain reaction.

## Data Availability

The data presented in this study are available on request from the corresponding author.

## Supplementary Materials

The following are available online at https://www.mdpi.com/article/10.3390/v13050838/s1, Figure S1: Maps of sampling sites indicating the proportion of PPRV N cELISA positive animals at each site: (a) buffalo interpretation 1 (positive percentage inhibition (PI) < 50); (b) buffalo interpretation 2 (positive PI < 60); (c) Grant’s gazelle interpretation 1; (d) Grant’s gazelle interpretation 2. Table S1: Summary of serological results using interpretation E1 (positive PI < 50) and E2 (positive PI < 60). Figure S2: Number of animals sampled per site by species.

## Appendix A

### Capture and Immobilisation of Wild Animals

Purposive sampling, 2015–2016: On arrival at each sampling site, the first accessible juveniles or adults of the targeted species were identified. Calves were not sampled due to the potential presence of maternally-derived immunity. If the group of animals could be approached by vehicle to within 40 m then individual animals were darted intramuscularly using a long-range projector dart rifle (Daninject, Germany) to inject a chemical immobilising agent combination of etorphine hydrochloride (Immobilon, VetaPharma Ltd., Leeds, UK) and xylazine (Rompun, Bayer, Newbury, UK) prepared according to the size of the animal. If animals could not easily be approached, then the group of animals was driven into a drop-net capture system set up in the vicinity of the group. Net-captured animals were restrained and tranquilised using intravenous haloperidol (Haldol, Mercury Pharma International, London, UK). Once an animal was chemically or physically restrained, it was blindfolded, placed in sternal recumbency and its vital signs were assessed. When it was stable, the following samples were collected: whole blood for serum, conjunctival and nasal swabs and faeces. The sex, age (based on teeth eruption and wear, horn size and shape), body condition score (emaciated, thin, average, good, very good) and clinical observations of each animal were recorded. The global positioning system (GPS) coordinates were recorded for the sampling site. After sampling, the animals were released from the nets, or the immobilisation was chemically reversed using diprenorphine (Revivon, VetaPharma Ltd., Leeds, UK).

Cross-sectional survey of African buffalo and Grant’s gazelle, 2018–2019: Once a buffalo herd was identified, animals were selected for darting to obtain a range of ages (based on horn and body size), excluding calves. Animals were darted from a vehicle, using Daniject 1.5 cc or 3 cc darts containing 2–12 mg etorphine and 30–50 mg azaperone or 60–100 mg xylazine, depending on body size. The darted animal was followed until it became recumbent, when it was approached, blindfolded and placed in sternal recumbency. Samples were collected while monitoring breathing and pulse, after which the sedation was reversed with diprenorphine at three times the etorphine dose, and with naltrexone if there was respiratory depression. The animals were observed until they had recovered.

In MMNR and the western part of Serengeti NP, Grant’s gazelle were immobilised by darting with 3 mg etorphine and 30 mg azaperone, because they were sufficiently habituated to allow the team to approach within darting range. Sedation was reversed with 9 mg diprenorphine or 40 mg naltrexone. In the other parts of the study area in Tanzania, capture nets were used for Grant’s gazelle following the method described by Mdetele et al. [83]. A suitable place for setting up the nets was identified at the sampling site, and vehicles were used to locate a herd and drive a group of animals into the net, where they were restrained and tranquilised with 3–4 mg etorphine and 15 mg azaperone. After tranquilisation, the animals were blindfolded and placed in sternal recumbency before sample collection. Each animal was then released and observed for any physical or locomotor difficulties. This process was repeated as necessary until the required sample size was collected at each point. Although the target number of animals to be sampled per site was 5, because of the variability in the numbers of animals captured during each drive, the actual numbers sampled per site varied from 1–9.

A number of animals of other species were also captured and sampled during the course of the survey. Initially, a cross-sectional sample of impala was also planned, but several attempts to capture impala showed that darting this species was very difficult because the flight distance was very long (at least 100 m), and therefore, sampling of impala was stopped after the first field mission. However, 14 animals were sampled from 3 sites in the western part of the Serengeti NP. In addition, during net capture of Grant’s gazelle in NCA, herds of Grant’s gazelle were sometimes grazing together with Thomson’s gazelle, and therefore, some Thomson’s gazelle were captured in nets together with Grant’s gazelle. Nine Thomson’s gazelle were opportunistically sampled at three sites.

## Appendix B

### Sample Processing and Storage

All the samples were labelled with the date of collection and a unique identification number. During the 2015–2016 study, all samples were placed immediately into a cool box with ice packs for transportation by vehicle to the base location. Blood samples were left to clot and then the serum removed and put into cryovials later the same day. All samples were stored at −20 °C until the fieldwork was completed, when they were taken in cool boxes with ice packs to TAWIRI laboratory in Arusha or to KWS in Nairobi, Kenya, where they were stored at −80 °C. Sera and swabs were shipped in dry ice to the Pirbright Institute, UK, while faeces samples were shipped in dry ice to CIRAD, France. The samples were stored at −80 °C until analysed.

During the 2018–2019 studies, whole blood samples were placed in a cool box with ice packs while all other samples (swabs and faeces) were put into cryovials and placed immediately into liquid nitrogen. The blood samples were allowed to clot, then the serum was removed and put into cryovials later the same day and placed into liquid nitrogen. Swab, faeces and serum samples were stored in liquid nitrogen until they were transported to Sokoine University of Agriculture (SUA), Tanzania, or to KWS in Nairobi, Kenya, where they were stored at −80 °C until analysis or shipment. Swabs were analysed at SUA, while serum samples were shipped in dry ice to the Pirbright Institute, UK, and faeces samples were shipped in dry ice to CIRAD, France.
