# Supplementary material for: Peste des Petits Ruminants Virus Infection at the Wildlife–Livestock Interface in the Greater Serengeti Ecosystem, 2015–2019
Source: Viruses. 2021 May 6;13(5):838. doi: 10.3390/v13050838 (PMC8148116; doi:10.3390/v13050838)
Supplement: Supplementary file 1 [file viruses-13-00838-s001.zip › viruses-1160873-supplementary.pdf]

Supplementary Material

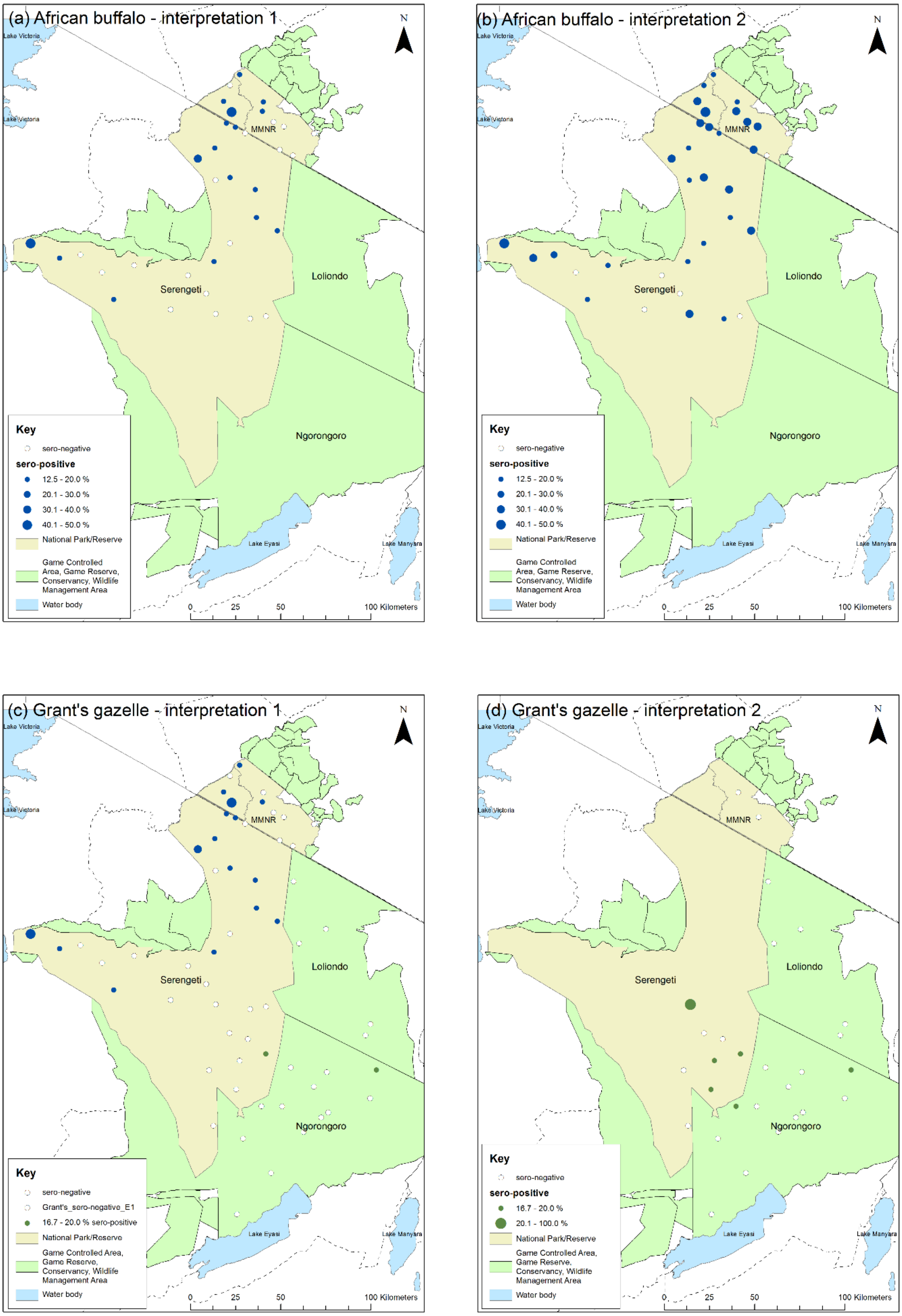

Figure S1 Maps of sampling sites indicating the proportion of PPRV N cELISA positive animals at each site: **(a)** buffalo interpretation 1 (positive percentage inhibition (PI) <50); **(b)** buffalo interpretation 2 (positive PI <60); **(c)** Grant's gazelle interpretation 1; **(d)** Grant's gazelle interpretation 2.

Table S1 Summary of serological results using interpretation 1 (positive PI <50) and interpretation 2 (positive PI <60)

			Interpretation 1		Interpretation 2	
**Variable**	**Category**	**Number sampled**	**Number positive (%, 95% CI)**	**Odds ratio (95% CI) Wald test P value**	**Number positive (%, 95% CI)**	**Odds ratio (95% CI) Wald test P value**
Species	African buffalo	191	23 (12.04, 7.40-16.69)	9.52 (2.13-42.57) p=0.003	49 (25.65, 19.42-31.89)	7.65 (3.17-18.44) p=<0.001
	Grant's gazelle	139	2 (1.44, -0.55-3.43)	reference	6 (4.32, 0.91-7.72)	Reference
Country	Kenya	85	10 (11.76, 4.85-18.68)	2.07 (0.73-5.85) p=0.17	24 (28.24, 18.57-37.90)	2.73 (1.47-5.06) p=0.001
	Tanzania	245	15 (6.12, 3.10-9.14)	reference	31 (12.65, 8.47-16.84)	reference
Protected area	Eastern MMNR	37	2 (5.41, 2.01-12.82)	0.55 (0.11-2.86) p = 0.483	9 (24.32, 10.26-38.39)	1.39 (0.59-3.25) p=0.45
	Mara Triangle	48	8 (16.67, 5.97-27.36)	2.05 (0.68-6.13) p=0.201	15 (31.25, 17.95-44.55)	1.96 (0.94-4.07) p=0.072
	Serengeti NP	154	14 (9.09, 4.52-13.66)	reference	29 (18.83, 12.61-25.05)	reference
	NCA	67	1 (1.49, 1.44-4.43)	0.15 (0.18-1.20) p=0.073	2 (2.99, 1.14-7.11)	0.13 (0.03-0.57) p=0.007
	Loliondo	24	0 (0)	-	0 (0)	-
Age*	0.5-<1	6	0 (0)	1.19 per 1 year increase (1.04-1.37) p=0.011	0 (0)	1.11 per 1 year increase (1.01-1.23) p=0.029
	1-<2	35	0 (0)		3 (8.57)	
	2-<3	41	3 (7.32)		7 (17.07)	
	3-<4	65	3 (4.62)		10 (15.38)	
	4-<5	46	0 (0)		3 (6.52)	
	5-<6	21	1 (4.76)		2 (9.52)	
	6-<7	29	6 (20.69)		10 (34.48)	
	7-<8	17	3 (17.65)		4 (23.53)	
	8-<9	25	4 (16.00)		6 (24.00)	
	9-<10	5	1 (20.00)		1 (20.0)	
	10-<11	33	3 (9.09)		7 (21.21)	
	11-<12	2	1 (50.00)		1 (50.0)	
	12-<13	1	0 (0)		0 (0)	
	13-<14	1	0 (0)		0 (0)	
	14+	3	0 (0)		1 (33.33)	
Age category	Young	6	0 (0)	-	0 (0)	0
	Sub-adult	70	3 (4.29, -0.52-9.08)	0.52 (0.14-1.90) p=0.319	10 (14.29, 6.00-22.57)	0.86 (0.39-1.88) p=0.707
	Adult	213	17 (7.98, 4.32-11.64)	Reference	35 (16.43, 11.43-21.44)	Reference
	aged	41	5 (12.20, 2.02-22.37)	1.50 (0.47-4.72) p=0.491	10 (24.39, 11.03-37.75)	1.58 (0.68-3.66) p=0.283
Sex	Female	168	10 (5.95, 2.35-9.55)	Reference	26 (15.48, 10.00-20.98)	Reference
	Male	162	15 (9.26, 4.77-13.75)	1.51 (0.62-3.67) p=0.368	29 (17.90, 11.96-23.84)	1.14 (0.62-2.11) p=0.678
Herd size	Range 1-900	Median 50	Mean herd size if sero-positive 201.84 (SD 210.68), if sero-negative 118.56 (SD 164.59) t-test p value 0.018	1.002 per 1 animal increase (1.000-1.005) p=0.048	Mean herd size if sero-positive 187.04 (SD 197.55), if sero-negative 112.44 (SD 160.95) t-test p value 0.0028	1.002 per 1 animal increase (1.001-1.004) p=0.009
Proximity to livestock	<10 km	217	14 (6.45, 3.16-9.74)	0.76 (0.28-2.11) p=0.602	31 (14.29, 9.60-18.97)	0.61 (0.31-1.18) p=0.143
	>10 km	113	11 (9.73, 4.22-15.24)	reference	24 (21.24, 13.64-28.84)	Reference

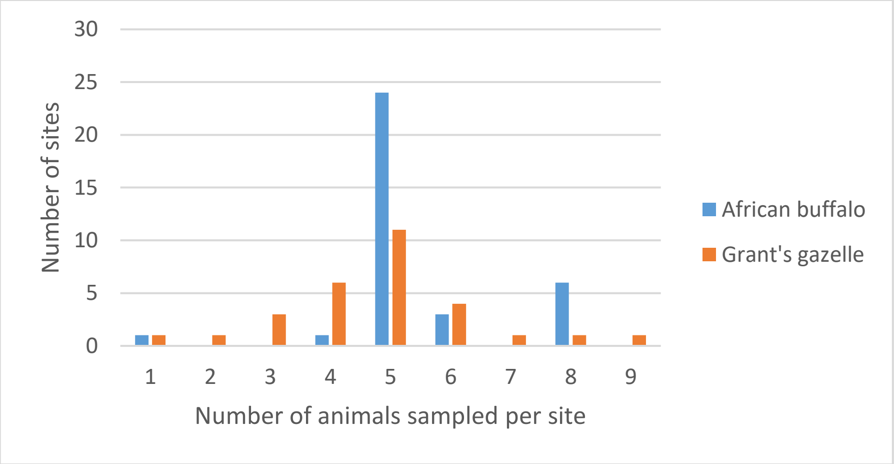

Figure S2. Number of animals sampled per site by species
